# The Efficacy and Safety of Abaloparatide‐SC in Men With Osteoporosis: A Randomized Clinical Trial

**DOI:** 10.1002/jbmr.4719

**Published:** 2022-10-18

**Authors:** Edward Czerwinski, Jose Cardona, Rafal Plebanski, Chris Recknor, Tamara Vokes, Kenneth G Saag, Neil Binkley, E Michael Lewiecki, Jonathan Adachi, Dorota Knychas, David Kendler, Eric Orwoll, Yinzhong Chen, Leny Pearman, Y Heather Li, Bruce Mitlak

**Affiliations:** ^1^ Krakow Medical Center Krakow Poland; ^2^ Indago Research & Health Center Hialeah FL USA; ^3^ Clinic of Healthy Bones Londz Poland; ^4^ Center for Advanced Research & Education Gainesville GA USA; ^5^ The University of Chicago Chicago IL USA; ^6^ The University of Alabama at Birmingham Birmingham AL USA; ^7^ University of Wisconsin Osteoporosis Clinical Research Program Madison WI USA; ^8^ New Mexico Clinical Research & Osteoporosis Center Albuquerque NM USA; ^9^ McMaster University Hamilton Ontario Canada; ^10^ Synexus Warsaw Medical Center Warsaw Poland; ^11^ University of British Columbia Vancouver British Columbia Canada; ^12^ Oregon Health & Science University Portland OR USA; ^13^ Radius Health, Inc. Boston MA USA

**Keywords:** ABALOPARATIDE, MEN, OSTEOPOROSIS, FRACTURE, BONE MINERAL DENSITY

## Abstract

Abaloparatide significantly increased bone mineral density (BMD) in women with postmenopausal osteoporosis and decreased risk of vertebral, nonvertebral, and clinical fractures compared with placebo. The Abaloparatide for the Treatment of Men with Osteoporosis (ATOM; NCT03512262) study evaluated the efficacy and safety of abaloparatide compared with placebo in men. Eligible men aged 40 to 85 years with osteoporosis were randomized 2:1 to daily subcutaneous injections of abaloparatide 80 μg or placebo for 12 months. The primary endpoint was change from baseline in lumbar spine BMD. Key secondary endpoints included BMD change from baseline at the total hip and femoral neck. A total of 228 men were randomized (abaloparatide, *n* = 149; placebo, *n* = 79). Baseline characteristics were similar across treatment groups (mean age, 68.3 years; mean lumbar spine BMD *T*‐score, −2.1). At 12 months, BMD gains were greater with abaloparatide compared with placebo at the lumbar spine (least squares mean percentage change [standard error]: 8.48 [0.54] versus 1.17 [0.72]), total hip (2.14 [0.27] versus 0.01 [0.35]), and femoral neck (2.98 [0.34] versus 0.15 [0.45]) (all *p* < 0.0001). The most common (≥5%) treatment‐emergent adverse events were injection site reaction, dizziness, nasopharyngitis, arthralgia, bronchitis, hypertension, and headache. During 12 months of abaloparatide treatment, men with osteoporosis exhibited rapid and significant improvements in BMD with a safety profile consistent with previous studies. These results suggest abaloparatide can be considered as an effective anabolic treatment option for men with osteoporosis. © 2022 Radius Health Inc and The Authors. *Journal of Bone and Mineral Research* published by Wiley Periodicals LLC on behalf of American Society for Bone and Mineral Research (ASBMR).

## Graphical abstract

Change from baseline in bone mineral density. *p < 0.0001. In men with osteoporosis, least squares mean percentage change (standard error) from baseline in lumbar spine BMD at 12 months was 8.48% (0.54%) with abaloparatide compared with 1.17% (0.72%) with placebo (*p* < 0.0001).

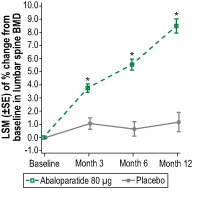

## Introduction

Osteoporosis in men is an important but underappreciated public health problem.^(^ ^1^ ^)^ Approximately one in four men over 50 years of age will incur a fragility fracture in their lifetime, with men accounting for up to 30% of the societal burden of osteoporosis and fractures.^(^ ^1^, ^2^, ^3^, ^4^ ^)^ Although osteoporosis prevalence is lower in men than in women, men have greater fracture‐related morbidity and mortality.^(^ ^5^ ^)^ Further, lower proportions of men initiate an appropriate osteoporosis treatment after a fracture, which contributes to consequent morbidity and mortality.^(^ ^6^ ^)^ A retrospective review of Medicare claims data from 2012–2016 showed that the rates of testing and/or treatment of osteoporosis among patients ≥65 years of age with fractures were lower in men (5.7%) than in women (12.1%).^(^ ^7^ ^)^ Similarly, a study with a 1‐ to 5‐year follow‐up after hip fracture found fewer men (27%) than women (71%) treated for osteoporosis.^(^ ^6^ ^)^ A more recent study showed that only 24% of patients (men and women combined) were treated after hip fracture and also reported that the proportion of men treated within 1 year after hip fracture was lower than women.^(^ ^8^ ^)^ Thus, the widely appreciated care gap after an osteoporosis‐related fracture is even greater in men than in women.

Currently, medications approved for the treatment of osteoporosis in men include bisphosphonates (alendronate, risedronate, and zoledronic acid), denosumab, and teriparatide.^(^ ^9^ ^)^ Because the prevalence of osteoporosis and fractures is higher in women than in men, effectiveness of available osteoporosis treatments has been more extensively studied in women. In part because of the underlying differences in testosterone and estrogen levels and their changes with aging, it is unclear whether treatments that are effective in women are equally so in men.^(^ ^10^, ^11^ ^)^ Thus, the US Preventative Services Task Force has indicated that more trials for the treatment of osteoporosis that focus on or include men are needed.^(^ ^5^ ^)^

In the Abaloparatide Comparator Trial in Vertebral Endpoints (ACTIVE), abaloparatide increased bone mineral density (BMD) in women with postmenopausal osteoporosis, decreased the risk of vertebral, nonvertebral, and clinical fractures compared with placebo, and decreased the risk of major osteoporotic fracture compared with teriparatide and placebo.^(^ ^12^ ^)^

The primary objective of the Abaloparatide for the Treatment of Men with Osteoporosis (ATOM) study was to evaluate the efficacy and the safety of abaloparatide compared with placebo for osteoporosis treatment in men.

## Materials and Methods

### Study design

**Fig. 1:**
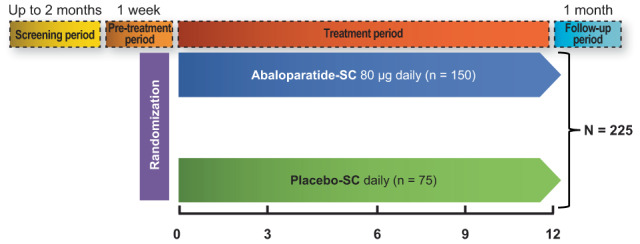
Study design. SC = subcutaneously. The numbers shown are the planned subjects to be randomized.

ATOM (NCT03512262), a randomized, double‐blind, placebo‐controlled, multicenter phase 3 study evaluating the efficacy and safety of abaloparatide compared with placebo for osteoporosis treatment in men, was conducted from May 3, 2018, to September 8, 2021. After a 1‐week pretreatment period, eligible volunteers were randomized 2:1 to receive daily subcutaneous (SC) injections of abaloparatide 80 μg or placebo SC for 12 months (Fig. 1). The study was conducted in accordance with the International Conference on Harmonization, the Declaration of Helsinki (2013), and applicable local regulations. Local institutional or central internal review boards (IRBs) (for some countries) were used to obtain approval from all institutions. All participants provided informed written consent to participate in the study.

### Participants

Men aged 40 to 85 years with primary osteoporosis or osteoporosis associated with hypogonadism were eligible to participate in the study. BMD *T*‐scores ≤ −2.5 and > −3.5 at the lumbar spine, total hip, or femoral neck (based upon the male reference database) or ≤ −1.5 and with radiologic vertebral fracture at screening or history of low‐trauma nonvertebral fracture in the past 5 years were required. Men >65 years of age who did not meet fracture criteria but who had BMD *T*‐scores ≤ −2.0 were also eligible. Hypogonadism at study entry was based on medical history or the laboratory reference range for serum testosterone at screening. Stable doses of androgen replacement therapy for at least 12 months were required. Good general health with a body mass index of 18.5 to 33 kg/m^2^ and a 25‐hydroxyvitamin D level of ≥20 ng/mL were also required.

Men with a history of fragility fracture within the past 12 months, >2 moderate vertebral fractures, or <2 radiologically evaluable lumbar vertebrae within L1 to L4 were excluded. Other exclusion criteria were prior treatment with parathyroid hormone (PTH)‐ or parathyroid hormone‐related peptide (PTHrP)‐derived drugs, intravenous bisphosphonates at any time, oral bisphosphonates within the past 3 years, denosumab within the past 18 months, or calcitonin or tibolone within the past 6 months. Men with serum levels of calcium (albumin corrected), PTH, thyroid‐stimulating hormone (TSH), phosphorus, and alkaline phosphatase outside the normal range and men with clinical signs of hypogonadism or low testosterone at screening but who had not yet initiated treatment were not eligible. Men with bone disorders (eg, Paget's disease) other than osteoporosis, a history of prior external beam or implant radiotherapy to the bone, any cancer in the past 5 years (other than basal cell or squamous cell cancer of the skin), or osteosarcoma at any time were also ineligible.

### Endpoints

The primary efficacy endpoint was percentage change from baseline in lumbar spine BMD by dual‐energy X‐ray absorptiometry (DXA) at 12 months. Key secondary endpoints were percentage change from baseline in total hip and femoral neck BMD at 12 months. Additional secondary endpoints included percentage change from baseline in BMD at 3 and 6 months for lumbar spine, total hip, and femoral neck; percentage change in BMD for the ultradistal radius and one‐third radius at 3, 6, and 12 months; and the incidence of new clinical fractures at 12 months. DXA scans were centrally adjudicated (BioClinica Medical Imaging, Princeton, NJ, USA). Clinical assessments for fracture were obtained on day 1 and at months 1, 3, 6, 9, and 12. Radiographs were read locally, and reports were reviewed at a central location. Lateral spine radiographs obtained at screening were graded according to the Genant semiquantitative scoring method for vertebral fractures (VSQ).^(^ ^13^ ^)^ Incident vertebral fractures were recorded with a VSQ score increase of ≥1.^(^ ^13^ ^)^ For clinical fractures at other anatomic locations (ie, wrist, hip, rib, etc.), radiographs were obtained at follow‐up visits as clinically warranted.

Additional efficacy endpoints included the change from baseline in serum procollagen type I N‐terminal propeptide (s‐PINP) and serum carboxy‐terminal cross‐linking telopeptide of type I collagen (s‐CTX), for which samples were collected pre‐dose at 1, 3, 6, and 12 months. Samples were batched and analyzed by Centre Académique de Recherche et d'Expérimentation en Santé (CARES) (University of Liège, Liège, Belgium).

Safety data were collected throughout the study and included treatment‐emergent adverse events (TEAEs), changes in vital signs (including orthostatic blood pressure at each blood pressure assessment), injection site reactions, and antibodies for immunogenicity testing. Laboratory tests (hematology, serum chemistry, and urinalysis) were conducted at screening and months 1, 6, and 12. Electrocardiograms were performed pre‐dose and 1 hour post‐dose on day 1 and at month 12. All adverse events (AEs) were coded using MedDRA.

### Statistical analysis

A sample size of 225 was determined to provide 99% power to detect a mean difference of 6.5% change from baseline in lumbar spine BMD at 12 months between treatment groups at an *α* level of 0.01, assuming a standard deviation of 6.0% and drop‐out rate of 10%. For efficacy analyses, the window convention was used to select data (ie, data could be selected within a designated timeframe for each visit). Primary and key secondary endpoints were analyzed by ANCOVA models using the intention‐to‐treat (ITT) population. Missing data were imputed using the wash‐out multiple imputation method.

To claim statistical significance at the 2‐sided level of 1%, the following three fixed‐sequence tests were performed in sequential order: (i) percentage change from baseline in BMD at the lumbar spine at 12 months; (ii) percentage change from baseline in BMD at the total hip at 12 months; (iii) percentage change from baseline in BMD at the femoral neck at 12 months. If the treatment difference was not statistically significant at the 1% level at any of the steps, all the subsequent comparisons following the fixed sequence could not be claimed statistically significant.

For the primary endpoint and secondary BMD endpoints, subgroup analyses were performed. Treatment groups were compared using ANCOVA models within subgroups defined by age, race, ethnicity, body mass index, geographic region, smoking status, BMD at baseline, fracture history, or s‐PINP levels at baseline.

Bone turnover markers were analyzed by ANCOVA models based on the ratio of the post‐baseline value relative to baseline at each visit, using a natural log transformation. Missing data were imputed using the wash‐out multiple imputation method.

A sensitivity analysis using the mixed model for repeated measures (MMRM) was performed to assess the impact of missing data. To investigate the effect of the COVID‐19 pandemic, several sensitivity analyses were performed, including subjects who completed or withdrew from the study before March 1, 2020, compared with subjects who completed or withdrew from the study on or after March 1, 2020. The nominal visit, excluding BMD measurements collected outside the allowable time period for data collection and excluding BMD measurements collected more than 30 days after the ideal visit days, were used.

Safety data were summarized descriptively.

## Results

**Fig. 2:**
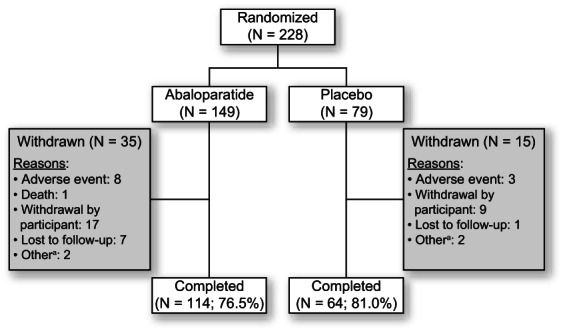
Participant disposition. ^a^Includes individual reasons such as incorrectly injecting study medication, starting an osteoporosis medication outside of the trial, and termination from the study based on physician decision.

A total of 228 men were randomized (abaloparatide, *n* = 149; placebo, *n* = 79) with 178 (78.1%) completing the study: 114 (76.5%) in the abaloparatide group and 64 (81.0%) in the placebo group (Fig. 2). The sensitivity analysis showed that the COVID‐19 pandemic did not appear to differentially affect study withdrawal rates or outcomes between groups.

Demographic and baseline characteristics were similar between treatment groups. Mean age was 68.3 years (abaloparatide, 68.5 years; placebo, 67.8 years) and mean lumbar spine BMD *T*‐score was −2.1 in each group (Table 1). Overall, 56.6% had a prior fracture (abaloparatide, 54.4%; placebo, 60.8%). Six (2.6%) men had primary hypogonadism at screening and 1 had secondary hypogonadism, all in the abaloparatide group.

**Table 1:** Participant Demographics and Clinical Characteristics (ITT Population)

Variable	Abaloparatide (*N* = 149)	Placebo (*N* = 79)	Total (*N* = 228)
Age (years), mean (SD)	68.5 (8.3)	67.8 (8.5)	68.3 (8.3)
Body mass index, mean (SD)	26.6 (3.5)	26.5 (3.5)	26.5 (3.5)
Race, *n* (%)			
White	140 (94.0)	76 (96.2)	216 (94.7)
Asian	8 (5.4)	1 (1.3)	9 (3.9)
Black or African American	0	1 (1.3)	1 (0.4)
Native Hawaiian or other Pacific Islander	1 (0.7)	0	1 (0.4)
Region, *n* (%)			
North America	76 (51.0)	43 (54.4)	119 (52.2)
Europe	73 (49.0)	36 (45.6)	109 (47.8)
Primary hypogonadism, *n* (%)	6 (4.0)	0	6 (2.6)
Secondary hypogonadism, *n* (%)	1 (0.7)	0	1 (0.4)
Total testosterone, mean (SD), nmol/L	16.3 (7.4)	16.2 (6.7)	16.3 (7.1)
*T*‐score, mean (SD)			
Femoral neck	−2.1 (0.6)	−2.2 (0.7)	−2.1 (0.6)
Total hip	−1.6 (0.7)	−1.7 (0.8)	−1.7 (0.7)
Lumbar spine	−2.1 (1.1)	−2.1 (1.2)	−2.1 (1.2)
Bone mineral density, mean (SD), g/cm^2^			
Femoral neck	0.698 (0.100)	0.691 (0.119)	0.696 (0.106)
Total hip	0.831 (0.093)	0.814 (0.116)	0.825 (0.102)
Lumbar spine	0.900 (0.145)	0.907 (0.162)	0.902 (0.151)
≥1 Prevalent vertebral fracture(s), *n* (%)	51 (34.2)	31 (39.2)	82 (36.0)
≥1 Prior fracture(s), *n* (%)	81 (54.4)	48 (60.8)	129 (56.6)
s‐PINP, ng/mL			
Mean (SD)	50.0 (16.8)	47.0 (20.5)	49.0 (18.2)
Median (min, max)	48.2 (14.4, 106.2)	41.3 (19.2, 127.2)	45.6 (14.4, 127.2)
s‐CTX, ng/mL			
Mean (SD)	0.360 (0.159)	0.336 (0.180)	0.351 (0.167)
Median (min, max)	0.327 (0.11, 1.04)	0.277 (0.11, 1.02)	0.312 (0.11, 1.04)

ITT = intention to treat; s‐CTX = serum carboxy‐terminal cross‐linking telopeptide of type I collagen; SD = standard deviation; s‐PINP = serum procollagen type I N‐terminal propeptide.

### Bone mineral density

**Fig. 3:**
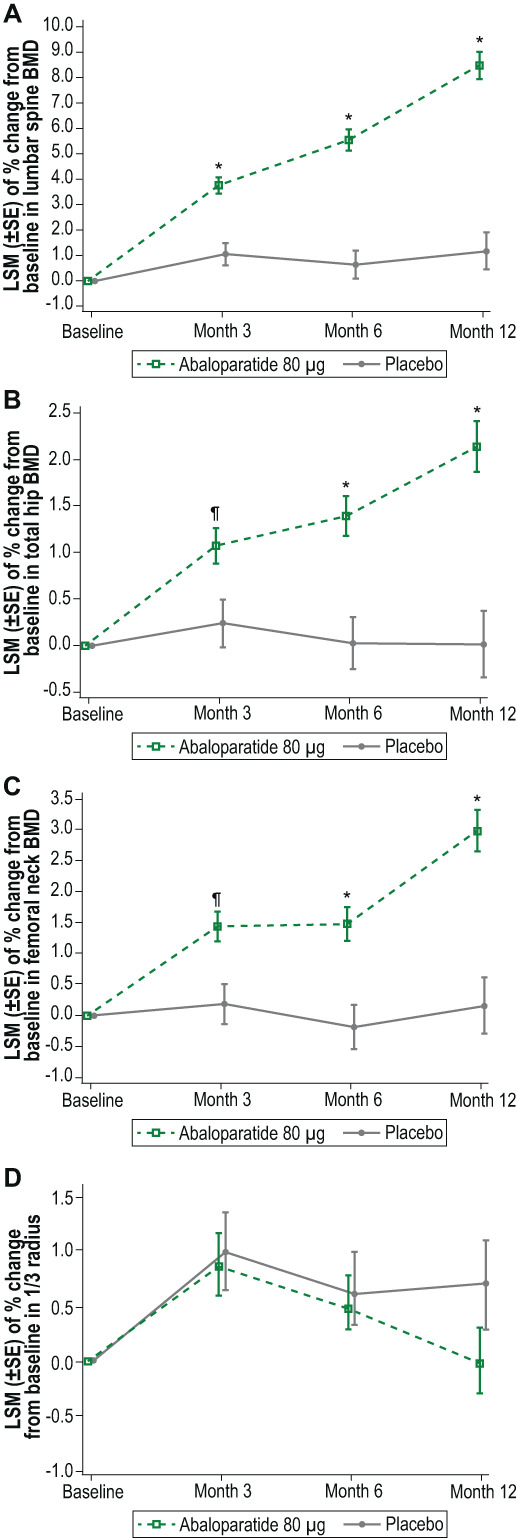
Change from baseline in bone mineral density. (*A*) Lumbar spine. (*B*) Total hip. (*C*) Femoral neck. (*D*) One‐third radius. ^¶^ *p* < 0.01; *p ≤ 0.0001. BMD = bone mineral density; LSM = least squares mean; SE = standard error.

For the primary outcome, least squares mean percentage change (standard error [SE]) from baseline in lumbar spine BMD at 12 months was 8.48% (0.54%) with abaloparatide compared with 1.17% (0.72%) with placebo (*p* < 0.0001) (Fig. 3*A*). Least squares mean percentage increases from baseline in BMD at 12 months at the total hip and femoral neck (Fig. 3*B*, *C*) were also statistically greater with abaloparatide (total hip: 2.14% [0.27%]; femoral neck: 2.98% [0.34%]) compared with placebo (total hip: 0.01% [0.35%]; femoral neck: 0.15% [0.45%]; all *p* < 0.0001). BMD gains at the lumbar spine, total hip, and femoral neck at 3 months were 3.76% (0.32%), 1.07% (0.19%), and 1.43% (0.24%), respectively, with abaloparatide compared with 1.06% (0.44%), 0.24% (0.25%), and 0.18% (0.32%) for placebo (all *p* < 0.01). At 6 months, BMD change at the lumbar spine, total hip, and femoral neck were 5.54% (0.41%), 1.39% (0.21%), and 1.48% (0.27%) with abaloparatide compared with 0.64% (0.55%), 0.03% (0.28%), and −0.19% (0.36%) for placebo (all *p* ≤ 0.0001).

Least squares mean BMD percentage change at the ultradistal radius at 3, 6, and 12 months were 1.28% (0.33%), 1.55% (0.36%), and 1.44% (0.42%), respectively, with abaloparatide, and −0.35% (0.45%), −0.32% (0.48%), −0.19% (0.57%) for placebo (all *p* < 0.05). However, BMD change compared with placebo at the one‐third radius was not statistically significant at any time point (abaloparatide: 3 months, 0.87% [0.28%]; 6 months, 0.48% [0.25%]; 12 months, −0.01% [0.33%]; placebo: 3 months, 0.99% [0.37%]; 6 months, 0.60% [0.32%]; 12 months, 0.71% [0.43%]) (Fig. 3*D*).

Lumbar spine, total hip, femoral neck, and ultradistal radius BMD results at month 12 were consistent in the abaloparatide group regardless of age, BMD at baseline, fracture history, or s‐PINP levels at baseline.

### Fracture incidence

Very few fractures occurred in either treatment group over the relatively short 12‐month study period. In the abaloparatide group, there was one new clinical fracture (forearm) and in the placebo group there were three patients with a new clinical fracture (spine, clavicle, and knee).

### Bone turnover markers

**Fig. 4:**
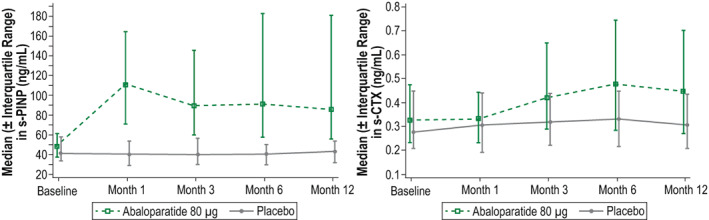
Median (±interquartile range) serum bone turnover marker levels. For s‐PINP, all *p* < 0.0001; for s‐CTX, *p* < 0.001 at months 3, 6, and 12. s‐CTX = serum carboxy‐terminal cross‐linking telopeptide of type I collagen; s‐PINP = serum procollagen type I N‐terminal propeptide.

In the abaloparatide group, median s‐PINP level peaked at month 1 (111.17 ng/mL) and was 85.7 ng/mL at month 12 (Fig. 4*A*). Geometric mean ratio relative to baseline was significantly greater than placebo at all time points (all *p* < 0.0001). Median s‐CTX level peaked at month 6 (0.476 ng/mL) and was 0.448 ng/mL at month 12 (Fig. 4*B*). Geometric mean ratio relative to baseline was significantly greater than placebo at months 3, 6, and 12 (all *p* < 0.001).

### Safety

The proportion of participants with any TEAE was similar between groups (abaloparatide, 117 [78.5%]; placebo, 57 [72.2%]) (Table 2). The most frequently reported TEAE was injection site erythema (abaloparatide, 19 [12.8%]; placebo, 4 [5.1%]). Serious TEAEs (abaloparatide, 8 [5.4%]; placebo, 4 [5.1%]) and TEAEs leading to study discontinuation (abaloparatide, 8 [5.4%]; placebo, 4 [5.1%]) were also similar between groups. For men treated with abaloparatide, dizziness (3 [2.0%]) was the most frequent TEAE leading to study discontinuation, with two of these events judged to be possibly related to study drug. One subject in the abaloparatide group had a fatal AE of non‐Hodgkin's lymphoma, which occurred 94 days after the last dose of abaloparatide treatment.

**Table 2:** Safety and Adverse Events (Safety Population)

	Abaloparatide (*n* = 149)	Placebo (*n* = 79)
Participants with any TEAEs, *n* (%)	117 (78.5)	57 (72.2)
Participants with any serious TEAEs, *n* (%)	8 (5.4)	4 (5.1)
TEAEs leading to deaths, *n* (%)^a^	0	0
TEAEs leading to study discontinuation, *n* (%)	8 (5.4)	4 (5.1)
TEAEs occurring in >5% of patients (abaloparatide arm), *n* (%)		
Injection site erythema	19 (12.8)	4 (5.1)
Nasopharyngitis	13 (8.7)	6 (7.6)
Dizziness	13 (8.7)	1 (1.3)
Arthralgia	10 (6.7)	1 (1.3)
Injection site swelling	10 (6.7)	0
Injection site pain	9 (6.0)	0
Headache	8 (5.4)	4 (5.1)
Hypertension	8 (5.4)	4 (5.1)
Bronchitis	8 (5.4)	1 (1.3)

AE = adverse event; TEAE = treatment‐emergent AE.

^a^ One fatal AE of non‐Hodgkin's lymphoma occurred 94 days after the last dose of abaloparatide treatment and was not a TEAE. The investigator assessed the event as not related to the study treatment.

Prior studies in women have suggested that orthostatic hypotension, hypercalcemia, and hypercalciuria may occur with abaloparatide treatment.^(^ ^14^ ^)^ Orthostatic hypotension was reported in no patients in the placebo group and in 2 (1.3%) patients treated with abaloparatide. Neither of these events was severe or serious. Hypercalcemia was reported in 2 (1.3%) men treated with abaloparatide. No hypercalcemia events were reported in the placebo group. Hypercalciuria was reported in both treatment groups (abaloparatide, 2 [1.3%]; placebo, 2 [2.5%]). The only patient to discontinue due to hypercalciuria was in the placebo group.

Mean changes in heart rate at 1 hour post‐dose were 5.7 beats/min (bpm) on day 1 and 5.3 bpm on month 12 in the abaloparatide group compared with −1.3 bpm on day 1 and 0.6 bpm on month 12 in the placebo group. A consistent and low percentage of men had QT shifts (corrected by Fredericia; QTcF) over the course of the study. No difference between treatment groups was observed in the incidence of clinically important QTcF values, the number of men with shifts in QTcF, or in the overall assessment of electrocardiograms.

## Discussion

In the current study in men with osteoporosis, the use of abaloparatide for 12 months resulted in significant and rapid increases in BMD at the lumbar spine, total hip, and femoral neck compared with placebo. Statistically significant changes in bone turnover markers were observed in the abaloparatide group, consistent with observed BMD changes and with previously reported changes in bone turnover markers in women treated with abaloparatide.^(^ ^12^ ^)^ A subgroup analysis in the current study also showed that BMD increases at each anatomic location were consistent regardless of s‐PINP levels at baseline.

No new safety concerns with abaloparatide were observed and several of the most frequently reported AEs in men were also among the most frequent previously reported in the ACTIVE study in women (dizziness, arthralgia, upper respiratory tract infection, headache, hypertension, and nasopharyngitis).^(^ ^12^ ^)^

Although some recent guidelines have recommended age‐ and risk‐based osteoporosis screening for men,^(^ ^15^, ^16^, ^17^ ^)^ evidence for osteoporosis treatment efficacy in men is limited.^(^ ^18^ ^)^ Separate studies have demonstrated a decreased rate of new morphometric vertebral fractures with zoledronic acid and denosumab compared with placebo.^(^ ^19^, ^20^ ^)^ Compared with alfacalcidol, alendronate and risedronate have also decreased vertebral fractures,^(^ ^21^, ^22^ ^)^ and there are observational data to suggest that fracture risk is reduced with teriparatide.^(^ ^23^ ^)^ Additionally, men on androgen deprivation therapy for prostate cancer treated with denosumab sustained fewer vertebral fractures than men who received placebo.^(^ ^24^ ^)^ However, most studies that have been conducted in men have been too small to assess fracture outcomes. The majority of studies have relied on surrogate outcomes such as BMD and bone turnover markers to assess efficacy,^(^ ^18^ ^)^ an approach that is further supported by a more recent study showing the benefits of using surrogate threshold endpoints for BMD improvements by anatomic location for the prediction of fracture efficacy.^(^ ^25^ ^)^ Based on this evidence, alendronate, risedronate, zoledronic acid, denosumab, and the anabolic therapy teriparatide are approved for use in men in the United States.

The current study provides further evidence for the use of anabolic therapy in men. Although comparisons between studies should be done with caution, BMD improvements in the current study appear similar to those observed in men treated with denosumab and zoledronic acid.^(^ ^19^, ^24^ ^)^ Additionally, increases in BMD with abaloparatide exceeded surrogate threshold estimates of BMD increases for predicting the clinical effectiveness for fracture reduction at all anatomic locations evaluated.^(^ ^25^ ^)^ Rapid rises in BMD and serum bone turnover markers in the current study are consistent with a recent study of romosozumab in men^(^ ^26^ ^)^ and previous studies of anabolic treatments in women,^(^ ^12^, ^27^ ^)^ suggesting that antifracture efficacy for dual action and anabolic agents in women may be extrapolated to men.

Limitations include the short 12‐month duration of this study and the relatively small sample size. Because of the small number of clinical fractures in this study, no meaningful comparison could be made between groups in terms of fracture incidence outcomes. Although larger studies in men that include fracture endpoints and active comparators would be informative, the use of BMD changes at the total hip has been proposed as a surrogate for anti‐fracture efficacy.^(^ ^18^ ^)^ The similar BMD and bone turnover marker outcomes between men in this study and those previously reported in women^(^ ^12^ ^)^ suggest that abaloparatide may provide an effective treatment option in men.

Abaloparatide rapidly improved lumbar spine and proximal femur BMD, suggesting that it may provide an effective treatment option for men with osteoporosis. Among men with osteoporosis, the self‐administration of abaloparatide was generally safe and well tolerated.

## Disclosures

EC reports travel support from Amgen and institutional funding from Radius Health, Inc., to conduct this study. JC and RP received institutional funding from Radius to conduct this study. CR has received research support and grants from Amgen, CytoDyn, Eli Lilly, Genentech, Integra, Novartis, Radius, Roche, and Senhwa and institutional funding from Radius to conduct this study. TV reports consulting fees and study funding from Takeda, study funding from Ascendis, and consulting and speaker fees from Radius outside of this work, and institutional funding from Radius to conduct this study. KGS has received research support from Horizon, Sobi, and Shanton and consulting fees from Arthrosi, Atom Bioscience, Horizon, Inflazome, L.G. Pharma, Mallinckrodt, Takeda, and Sobi pharmaceuticals and institutional funding from Radius to conduct this study. NCB reports consulting fees from Amgen and institutional funding from Radius to conduct this study. EML reports consulting fees and study funding from Amgen and Radius outside of this work and institutional funding from Radius to conduct this study. JA has provided consultancy to Amgen, Gilead, and Paladin Pharma; received research funding from Amgen and Radius; is an advisory board member of Amgen and Gilead; and participated in speaker bureaus for Amgen and Gilead. DKn received institutional funding from Radius to conduct this study. DKe reports speaker and consulting fees from Amgen, consulting fees from Paladin Pharma and Biosynt, and institutional funding from Radius to conduct this study. EO reports research support from Mereo, consulting fees from Amgen, Sanofi, Biogen, Bayer, NextCure, and Ultragenyx, and consulting fees from Radius in relation to this study. YC is a former employee of and holds equity in Radius. LP, YHL, and BM are employees of and hold equity in Radius.

## Author Contributions

**Edward Czerwinski:** Investigation; resources; writing – review and editing. **Jose Cardona:** Investigation; resources; writing – review and editing. **Rafal Plebanski:** Investigation; resources; writing – review and editing. **Chris Recknor:** Investigation; resources; writing – review and editing. **Tamara J. Vokes:** Investigation; resources; writing – review and editing. **Kenneth G Saag:** Investigation; resources; writing – review and editing. **Neil Binkley:** Investigation; resources; writing – review and editing. **E Michael Lewiecki:** Investigation; resources; writing – review and editing. **Jonathan Adachi:** Investigation; resources; writing – review and editing. **Dorota Knychas:** Investigation; resources; writing – review and editing. **David Kendler:** Investigation; resources; writing – review and editing. **Eric Orwoll:** Conceptualization; visualization; writing – review and editing. **Yinzhong Chen:** Data curation; formal analysis; resources; visualization; writing – review and editing. **Leny Pearman:** Resources; writing – review and editing. **Y Heather Li:** Resources; writing – review and editing. **Bruce Mitlak:** Conceptualization; funding acquisition; methodology; resources; supervision; visualization; writing – review and editing.

### Peer Review

The peer review history for this article is available at https://publons.com/publon/10.1002/jbmr.4719.

## Data Availability

Data that underlie the results reported in a published article may be requested for further research 6 months after completion of FDA or EMA regulatory review of a marketing application (if applicable) or 18 months after trial completion (whichever is latest). Radius will review requests individually to determine whether (i) the requests are legitimate and relevant and meet sound scientific research principles, and (ii) are within the scope of the participants' informed consent. Prior to making data available, requestors will be required to agree in writing to certain obligations, including without limitation, compliance with applicable privacy and other laws and regulations. Proposals should be directed to info@radiuspharm.com.
